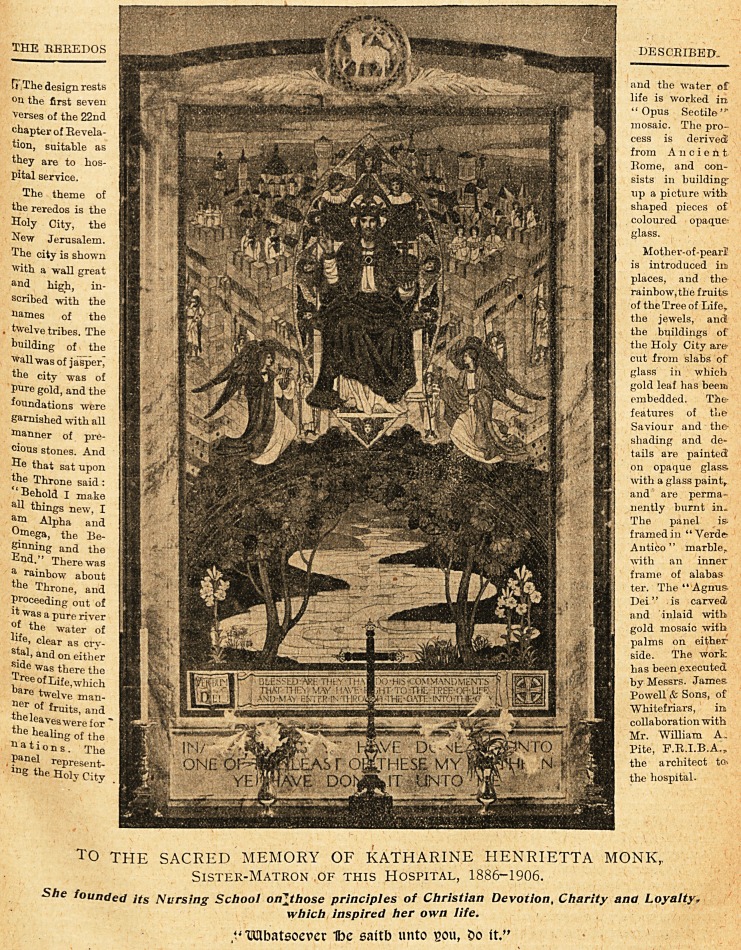# Reredos in King's College Hospital Chapel

**Published:** 1917-11-17

**Authors:** 


					November 17, 1917. THE HOSPITAL 147
SISTER-MATRON KATHARINE MONK
IRcre&os in Iking's College Ibospital Cbapel,
THE REREDOS
R The design rests
?n the first seven
"verses of the 22nd
chapter of Revela-
tion, suitable as
they are to hos-
pital service.
The theme of
the reredos is the
Holy City, the
New Jerusalem.
The city is shown
with a wall great
and high, in-
scribed with the
Raines of the
, twelve tribes. The
building of the
Wall was of jasper,
the city was of
pure gold, and the
foundations were
garnished with all
banner of pre-
cious stones. And
He that sat upon
the Throne said:
Behold I make
aU things new, I
an* Alpha and
?mega, the Be-
ginning and the
End." There was
a rainbow about
tbe Throne, and
Proceeding out of
Jt Was a pure river
of the water of
e> clear as cry-
stal, and on either
side was there the
^ree of Life, which
are twelve man-
ner of fruitSj and
the leaves were for'
the healing of the
Nations. The
, ?anel represent-
lng the Holy City
DESCRIBED.
and the water of
life is worked in
"Opus Sectile ""
mosaic. The pro-
cess is derived!
from Ancient
Rome, and con-
sists in building'
tip a picture with
shaped pieces of
coloured opaque:
glass.
Mother-of-pearl
is introduced ire
places, and the
rainbow,the fruits
of the Tree of Life,
the jewels, and
the buildings of
the Holy City are
cut from slabs of
glass in which
gold leaf has beem
embedded. Tie-
features of the
Saviour and the-
shading and de-
tails are painted
on opaque glass
with a glass paint,
and are perma-
nently burnt in.
The panel is
framed in "Verde-
Antico " marble,
with an inner
frame of alabas
ter. The "Agnus
Dei" is carved
and inlaid with
gold mosaic with
palms on either
side. The work
has been executed
by Messrs. James.
Powell & Sons, of
Whitefriars, in
collaboration with
Mr. "William A.
Pite, F.R.I.B.A.,
the architect to-,
the hospital.
the beredos
!!de was there the
^ree of Life, which
are twelve man-
"er ?f fruits, and
thelei
DESCRIBED.
shaped pieces of
coloured opaque;
glass.
the reredos is the
Holy City, the
New Jerusalem.
The city is shown
with a wall great
and high, in-
scribed with the
names of the
twelve tribes. The
building of the
"wall was of jasper,
the city was of
pure gold, and the
foundations were
garnished with all
manner of pre-
cious stones. And
He that sat upon
the Throne said:
'' Behold I make
all things new, I
am Alpha and
Omega, the Be-
ginning and the
End." There was
a rainbow about
the Throne, and
proceeding out of
it was a pure river
Se^clearaTcry- ? R? ^ ^ ^
stal, and on either MB WK
Mother-of-pearl!
is introduced iis
places, and the
rainbow,the fruits
of the Tree of Life,
the jewels, and
the buildings of
the Holy City are-
cut from slabs of
glass in which
gold leaf has been*
embedded. The-
features of the
Saviour and the-
shading and de-
tails are painted
on opaque glass,
with a glass paint,
and are perma-
nently burnt ill-
The panel is
framed in "Verde
Antico " marble,
with an inner
frame of alabas
ter. The " Agnus
Dei" is carved
and inlaid with
gold mosaic with
palms on either
has been executed
by Messrs. James.
Powell & Sons, of
Whitefriars, in
T0 THE SACRED MEMORY OF KATHARINE HENRIETTA MONK,
Sister-Matron of this Hospital, 1886-1906.
She founded its Nursing School onjthose principles of Christian Devotion, Charity and Loyalty,
which inspired her own life.
VMbatsoever 1be sattb unto sou, Do it."

				

## Figures and Tables

**Figure f1:**